# Prevention and Rehabilitation of the Athletic Hamstring Injury

**DOI:** 10.1016/j.asmr.2024.101021

**Published:** 2024-10-10

**Authors:** Adriana Geraci, Delaney Mahon, Eric Hu, Jesus E. Cervantes, Shane J. Nho

**Affiliations:** a Department of Physical Therapy, Midwest Orthopaedics at Rush, Rush University Medical Center, Chicago, IL, U.S.A.; b Section of Young Adult Hip Surgery, Division of Sports Medicine, Department of Orthopedic Surgery, Rush Medical College of Rush University, Rush University Medical Center, Chicago, IL, U.S.A.

## Abstract

Hamstring injuries are a common occurrence in both recreational and competitive athletes. Professional athletes have an increased incidence of hamstring-related injuries because most of these injuries are often attributed to movements involving forceful and rapid hamstring lengthening. The incidence of hamstring injuries is particularly high in professional baseball players, likely due to the high-speed running and frequent transition from static standing to dynamic movements inherent to the sport itself. To improve an athlete’s ability to return to sport and minimize days off due to injury, a programmed rehabilitation program combining a range of training parameters that ensure the athlete can work near the limit of their capacity while concurrently allowing sufficient time for the injured tissue to heal is essential. The purpose of this article is to summarize the different features and evaluation of hamstring injuries in athletes, highlight evidence-based rehabilitation interventions, and identify preventative strategies to effectively and efficiently treat athletes, particularly baseball players, with hamstring injuries.

**Level of Evidence:**

Level V, expert opinion.

In a sporting context, the most common mechanism of a hamstring-related injury is high-speed running, followed by movements involving forceful and rapid hamstring lengthening, such as kicking.^1^^,^^2^ Professional sports have an increased incidence of hamstring-related injuries secondary to the high volume of transitions from static standing to high-speed running and sprinting.^2^^,^^3^ After an acute hamstring injury, it is essential to combine a range of training parameters to ensure that the athlete can work near the limit of his or her capacity while concurrently ensuring that sufficient time is allowed for the injured tissue to heal.^4^ The purpose of this article is to summarize the different features and evaluation of hamstring injuries in athletes, highlight evidence-based rehabilitation interventions, and identify preventative strategies to treat athletes, particularly baseball players, with hamstring injuries effectively and efficiently.

## Epidemiology of Hamstring Injuries

In professional athletes, hamstring-related injuries are common with an incidence rate of approximately 29% within the athletic community.^3^ Studies have shown that hamstring injuries peak between 16 and 25 years of age. In professional soccer leagues, younger athletes tend to have lower rates of injury with an average loss of play of 21 days.^5^ In the National Football League (NFL), a 10-year study revealed that 50% of all hamstring injuries occurred during preseason training, with the most injury prone positions being defensive secondaries and wide receivers.^6^

The incidence of hamstring injuries in professional baseball is higher than other professional sports with an injury rate of 1.09 per 1,000 athlete exposures in the Major League Baseball (MLB) and 1.17 per 1000 athlete-exposures in the Minor League Baseball (MiLB).^3^ An injury to a professional baseball player causing missed days and time on the disabled list is of large competitive and financial interest for professional baseball organizations.^7^ In the MLB, hamstring injuries are the fourth leading cause of days on the disabled list for all players, regardless of position, and the second leading cause of days on the disabled list for field position players.^7^ The high incidence of hamstring-related injuries within professional baseball appears to be increasing,^3^^,^^8^ thus demonstrating the importance of preventative measures and effective rehabilitative methods to reduce the risk of reinjury and minimize missed days.

## Anatomy and Pathophysiology

The hamstring is composed of 3 main muscles: the biceps femoris (long and short head), semitendinosus, and semimembranosus.^9^ The semitendinosus and long head of the biceps share a common origin, which is the ischial tuberosity.^9^ The long head of the biceps femoris arises from the medial facet of the ischial tuberosity and the short head of the biceps femoris arises from the lateral aspect of the linea aspera, lateral supracondylar line, and intermuscular septum.^9^ The semimembranosus originates more superiorly on the ischial tuberosity compared with the semitendinosus and biceps femoris.^9^

Hamstring injuries can occur from repetitive microscopic muscle damage or a single event exceeding the limits of the muscle-tendon unit.^1^ Some injuries may occur from a decline in tissue integrity due to repetitive damage.^1^ A macro-traumatic event can occur where there is a forceful and rapid hip flexion moment.^1^ Hamstring injuries can be categorized into 3 categories: active or passive high muscle-tendon unit forces, muscle tendon unit lengthening beyond moderate lengths, and high-velocity movements.^1^

Hamstring injuries are classified on a scale of 1 to 3: grade 1 injuries (hamstring strain) are defined as <5% muscle disruption, grade 2 injuries (partial hamstring tear) as >5% muscle disruption and no retraction of muscle ends, and grade 3 (complete hamstring tear) as complete discontinuity of muscle fibers with retraction of muscle ends.^3^

## Clinical Examination

### Subjective Interview

Athletes complaining of an acute onset of posterior thigh pain should be evaluated for a possible hamstring related injury.^10^ Some athletes may report pain accompanied by an audible or sensory pop sensation, but this is typically limited to high-grade injuries and may not always be present.^10^ It is important when taking a thorough subjective examination to ask the athlete if they have had a history of hamstring-related injury because this represents a significant risk of reinjury.^10^

### Objective Testing

Objective testing should include palpation examination, which should be performed with the athlete lying prone with knees in full extension. The practitioner may palpate the distance from the ischial tuberosity distally to the point of maximal pain provocation to identify the possible injury site.^10^ The point of maximal pain provocation and any defects in the muscle-tension unit should be documented to monitor throughout rehabilitation.^10^

Range of motion (ROM) and strength measurements should also be included in the clinical examination. Hip-flexion and knee-extension measurements should be evaluated, although pain may limit the accurate assessment of muscle length extensibility.^10^ Practitioners may use the active knee extension test (i.e., 90-90 straight leg test) to assess the hamstring in a position of maximal length. Comparison to the contralateral unaffected limb should be performed to track tolerance to maximal hamstring lengthening over treatment. Strength testing should include measurements of knee-flexion and hip-extension strength with the athlete in both prone and supine positions. It is highly recommended that clinical tools such as a handheld dynamometer, load cells, or force plates, be used to quantify muscle strength objectively. At the least, however, manual muscle testing should be performed in a standard manner if no other tools are available.^10^^,^^11^

Throughout the course of treatment, practitioners should monitor muscle extensibility and strength, as well as the level of pain reported by athletes on a visual analog scale (VAS). The integration of subjective measures and objective testing plays a crucial role in shaping treatment plans.

### Imaging

Other than subjective and objective clinical testing, magnetic resonance imaging (MRI) may also be used to diagnose and properly stage the location and extension of hamstring tissue damage.^10^ In addition, there are proposed MRI-based muscle-injury classifications to provide a more accurate return-to-sport diagnosis.^10^ Ultrasound may also be used to identify tendon thickening, an alteration in tendon echotexture, or entheseal bony irregularity at the cortical surface of the ischial tuberosity. However, a confident diagnosis of a partial tear can be difficult.^11^

### Grading

Hamstring injury grade can be classified based on either the traditional clinical presentation system or the MRI grading system for muscle injury.^12^ The traditional system classifies hamstring injuries as follows: grade 1 hamstring injuries may be associated with mild pain and loss of function, but no discernable disruption of fibers. Grade 2 hamstring injuries are associated with partial disruption of fibers. Grade 3 hamstring injuries are reserved for complete tears.^13^^,^^14^ The MRI grading system for muscular injury is as follows: grade 1 injuries are associated with T2 hyperintense signal surrounding a tendon or muscle, but absence of muscle fiber disruption. Grade 2 injuries involve T2 hyperintense signal surrounding or within a tendon or muscle with associated muscle fiber disruption less than half the width of the tendon/muscle. Grade 3 injuries involve extensive tendon retraction with disruption of muscle fibers less than half its width.^12^

## Risk Factors

### Position

There are multiple risk factors that contribute to the high incidence of hamstring injuries in athletes (Table 1). Regarding lower extremity positioning, forceful eccentric hamstring loading while in the fully lengthened position with the hip flexed and knee extended put the hamstring at risk of injury. While this motion is seen across various sports disciplines, the association between hamstring injuries and baseball has been widely studied. It has been suggested that the sport of baseball itself contributes to an inherent risk of hamstring injury secondary to the rapid switch from a static position to dynamic activity.^3^ A recent study found that most hamstring muscle strains occurred while base running, at a rate of 62% in the MLB and 70.6% of MiLB with fielding being the second most common activity.^15^ This is likely due to a stretch-type injury with the hamstrings contracting forcefully while lengthening to decelerate the limb in hip flexion and simultaneous knee extension.^7^^,^^16^^,^^17^ The run to first base from batting is unique due to the preceding truncal rotation, urgent acceleration without the ability to lead, and the demand to maximize speed as the player is directed to “run through first base.”^7^ This particular run path poses the highest risk for lower extremity muscular strain.^7^

**Table 1:** Risk Factors and of Hamstring Injury and Reinjury^17^^,^^19^^,^^25^, ^26^, ^27^

Age	Increasing age has been shown to be an independent risk factor for hamstring injuries.
Activity and position	Activities involving high intensity running and extensive hamstring contraction during hip flexion and knee extension are both independent risk factors.
Velocity	Running speed and hamstring muscle force have a positive correlation. In turn, heightened velocity raises the risk of hamstring injuries, particularly the biceps femoris.
Previous history of injury	Post–hamstring injury complications, including the development of scar tissue, decreased flexibility, eccentric strength reduction, and muscle atrophy, tend to alter lower limb biomechanics, heightening the risk of reinjury.
Knee extension deficit	Excessive tendon load during rapid eccentric contraction in the setting of poor flexibility and short hamstring fascicles has been shown to lead to increased hamstring injuries.
H/Q muscle strength ratio	A low H/Q strength ratio results in the quadricep muscles overpowering the mechanical limits of the hamstring, significantly increasing the risk of injury, especially when the concentric H/Q ratio is below 50%.

H/Q, hamstring-to-quadricep.

### Knee Extension Deficit

Another risk factor for hamstring injury is an active knee extension deficit. Peak hamstring lengthening occurs during the eccentric contraction of the hamstring in the late swing phase of the running gait cycle, and this rapid contraction paired with poor flexibility has contributed to an increase in hamstring-related injuries.^16^^,^^18^ Short hamstring fascicles, as found with poor flexibility and decreased hamstring muscle length, in conjunction with the eccentric contraction of the late swing phase contribute to an excessive load that the tendon cannot withstand leading to tissue failure and injury.^16^ One may use clinical findings of an active knee extension deficit to predict a poor hamstring length-tension relation contributing to risk of injury with running and/or sprinting in sport.^18^

### Previous History of Injury

One of the major risk factors for a hamstring injury is the history of a previous hamstring injury. In one study, reinjury rates were found to be as high as 20% in MLB and 8% in MiLB.^3^ Maladaptations as well as accumulated microscopic muscle damage after initial injury are thought to contribute to an athlete’s increased risk of reinjury.^17^ Maladaptations including formation of nonfunctional scar tissue after initial injury can decrease hamstring flexibility and influence the length-tension relationship, thus increasing the risk of reinjury when performing rapid deceleration movements while sprinting.^17^ This scar tissue formation is found evident in MRI for up to 1 year after return to sports, contributing to impaired mechanical properties of the hamstrings.^19^ In addition to muscular fiber changes, asymmetrical strength and loading mechanics may also contribute to increased risk of reinjury if not addressed in rehabilitation after initial injury.^17^

### Hamstring-to-Quadricep Muscle Strength Ratio

Another risk factor for hamstring injury in athletes, specifically baseball players, is a poor hamstring-to-quadricep strength ratio (H/Q). The hamstrings are thought to act as the “brakes” for deceleration during the late swing phase of running gait, and a low H/Q ratio may contribute to the quadricep muscles overpowering the mechanical limits of the hamstring.^17^ It has been found that players with a concentric H/Q strength ratio below 50.5% increased the risk of a hamstring strain injury by 3-fold.^20^

### Velocity

During high-speed running, the hamstrings are notably the most active, rapidly lengthening, and absorbing energy to decelerate the limb in preparation for foot contact during the terminal swing phase.^1^ This hamstring muscle force increases 1.3-fold as running velocity increases from 80% to 100% of maximum with the greatest muscle-tendon unit stretch, which is incurred by the long head of the biceps femoris.^1^ This increase in velocity contributes to an increase in the risk of a hamstring-related injury, most notably in the biceps femoris, which is the most injured hamstring muscle.^1^

## Rehabilitation Principles and Timeline of Recovery

One of the most frequently asked questions from players and coaches at all levels is when the athlete will be able to return to play. The answer to this question depends on multiple factors, including location of injury, grade of injury, surgical versus conservative management, and history of a previous injury. All these factors must be considered when determining clinical management. Although approach to rehabilitation is tailored according to injury location, severity, and patient/sport-specific goals of therapy, management generally includes physiotherapy with possible concomitant surgical intervention or injections of platelet-rich plasma.^2^^,^^4^ One study found that, on average, 16% of MLB and 10% of MiLB players missed more than 30 days in season after a hamstring related injury.^3^ The number of mean days missed also increased when players were managed surgically compared with conservatively.^3^ The main goal of a rehabilitation program after hamstring injury is to facilitate the athlete returning to sport at the highest possible performance level as fast as possible but with a minimal risk of reinjury.^4^

### Phases of Rehabilitation

**Fig 1:**
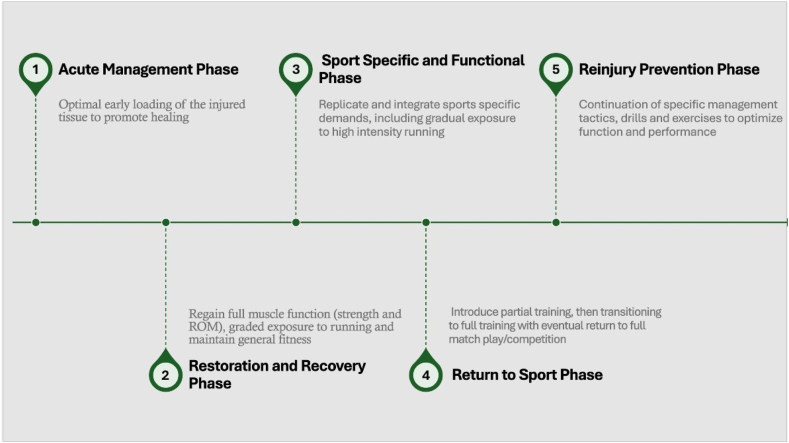
Phases of rehabilitation, including the (1) acute management phase, (2) restoration and recovery phase, (3) sport specific and functional phase, (4) return to sport phase, and (5) reinjury prevention phase. ROM, range of motion.

Phases of rehabilitation guide the clinician on how to progress the patient but should consider the patient’s deficits and presentation. It should be kept in mind that there are no distinct borders between these phases.^4^ Thus, rehabilitation can be considered as a process in which the phases will often overlap.^4^ Most commonly, rehabilitation can be broken down into 5 phases, including acute management, restoration and recovery, sport-specific, return to sport, and reinjury prevention (Fig 1).

### Eccentric Training

**Fig 2:**
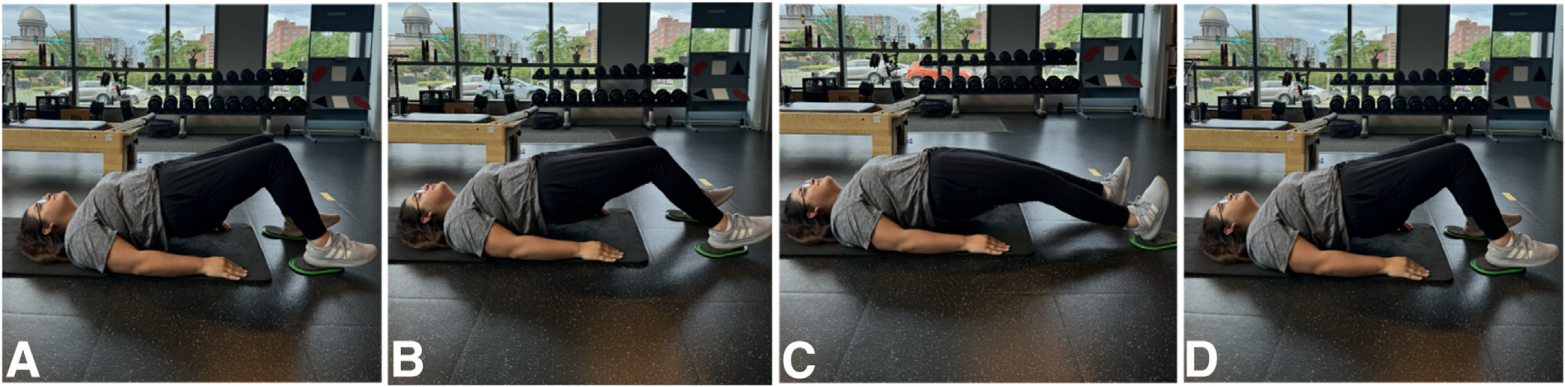
Submaximal eccentric hamstring slider. Images A through D depict the submaximal bilateral eccentric hamstring slider exercise. (A) Initially, the heels of both feet are positioned on top of sliders that allow smooth movement along the floor. The bottom is lifted off the ground, and both arms are positioned down the side to provide stability. (B) While maintaining tension, the athlete slides the heels away from the head while keeping the bottom from touching the floor, continuously engaging the posterior chain and hamstrings. (C) The feet are slid distally until full extension of the leg is achieved. (D) The feet are brought back into the same initial position through the same sliding motion, which represents 1 repetition.

Athletes fully compliant with an eccentric training program experienced fewer reinjuries and reduced strength deficits compared with noncompliant patients.^2^ Eccentric muscle exercises are defined as active lengthening of muscle fibers while contracting.^7^ Eccentric training enabled faster return to play (RTP) for elite soccer and track and field athletes compared with conventional training regardless of whether the injury was of sprinting or stretching type.^2^^,^^4^^,^^21^ Eccentric hamstring exercises are often avoided in the early stages of hamstring rehabilitation and only introduced after pain and between-limb strength deficits during isometric knee flexion have resolved.^1^ Nevertheless, eccentric loading can be safely progressed based on individual exercise performance, regardless of pain and between-limb strength deficits during isometric knee flexion after acute hamstring injury.^1^ For example, the submaximal bilateral eccentric hamstring slider exercise (Fig 2) can be introduced at the very start of hamstring injury rehabilitation, and when athletes can perform this exercise through full ROM, they can progress to a unilateral variation and begin the Nordic hamstring exercise.^1^ This progressive approach to eccentric loading has been shown to increase hamstring strength and the long head of the biceps femoris muscle fascicle length in relatively brief periods of rehabilitation after acute hamstring injury.^1^ Reinjury rate did not differ between eccentric and conventional rehabilitation protocols.^2^ However, athletes fully compliant with an eccentric training program experienced fewer reinjuries and reduced strength deficits compared with noncompliant patients.^2^

### Nordic Hamstring Curls

**Fig 3:**
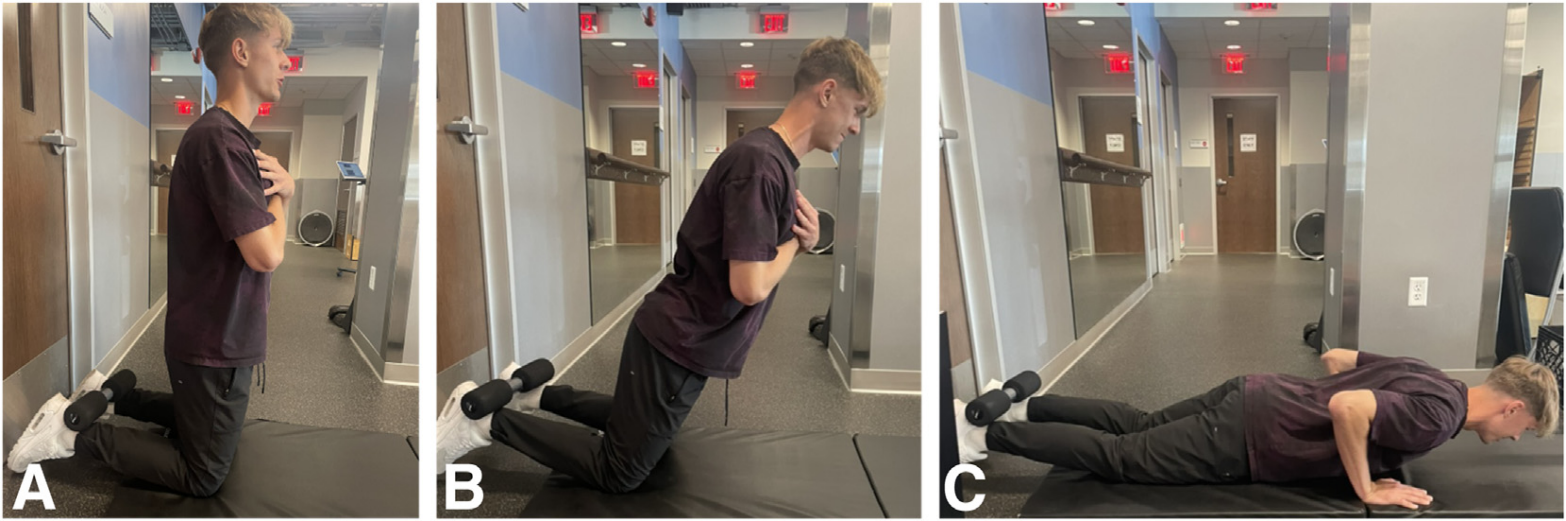
Nordic hamstring curl. Images A through C depict the Nordic hamstring curl. (A) The athlete starts with the heels supported superiorly by a foam bar and the knees bent at 90°. (B) The arms are crossed in front to isolate hamstring contraction. The athlete slowly leans forward, continuously resisting forward-falling motion. (C) Once achieving the goal of a near parallel position, the athlete can use their arms to push up back into the starting position for 1 repetition.

A detailed explanation is provided to ensure correct performance of the Nordic hamstring exercise (Fig 3). A thorough warm-up should be done before all exercises. The Nordic exercise can be performed either with a partner or using specialized equipment. The athlete begins the exercise on his knees with his torso perpendicular to the ground, while his partner/ankleholder applies pressure to his heels/ankles to ensure the athlete’s feet stay in contact with the ground throughout the motion of the exercise.^7^ The athlete then resists a forward-falling motion by eccentrically engaging the hamstring muscles.^7^ The athlete is instructed to resist forward-falling as long as possible, attempting to reach a position parallel to the floor. The athlete then allows his body to fall using his hands and arms to cushion the impact and quickly push up to the starting position.^7^ An overall average of 3.5 repetitions per week spread throughout the entire year was an adequate level of intervention that one would begin to see therapeutic benefit.^7^

### Return to Running

Neuromusculoskeletal models have demonstrated that peak hamstring force and stretch occur during the late-swing phase of the running gait cycle.^7^ It is at this point, when the hamstring muscles are generating tension while lengthening (eccentric contraction) to decelerate knee extension, when the hamstring muscle group is at high risk of injury.^7^ A progressive return to high-speed running and sprinting is likely the most important aspect of rehabilitation, given that it is fundamental to performance in many sports and a common hamstring injury mechanism.^1^ Stage 1 can be safely introduced after athletes can walk with minimal pain (e.g., pain ≤4 on a numeric rating scale ranging from 0 to 10), progressing from a slow jog (approximately 25% of maximal velocity) to moderate-speed running (approximately 50% of maximal velocity) as tolerated.^1^ When moderate-speed running is tolerated, athletes can gradually progress through stage 2 but should only advance to stage 3 when high-speed running (approximately 80% of maximal velocity) can be performed without pain to minimize the hamstring injury risk.^1^ During stage 3, progression toward maximal sprinting (100% of maximal velocity) should occur in relatively small increments (approximately 5%) to account for the substantial increase in negative (i.e., eccentric) work required by the hamstring at running intensities >80% of maximal velocity.^1^

## Return to Play Criteria

### Objective Measures Before Return to Sports

Pain, patient-reported outcomes, apprehension, and eccentric hamstring strength are all important objective measures that should be examined prior to returning to sport. Pain is measured by the VAS, which is a subjective measure for acute and chronic pain ranging from 0 to 10.^1^

The Functional Assessment Scale for Acute HSIs, a patient-reported outcome, is a questionnaire that has recently been developed as a disease-specific and self-administered questionnaire to grade the severity of symptoms (pain and function) in patients with hamstring injuries.^1^^,^^22^ HAGOS is another self-directed questionnaire used to provide details on the injury history, individual player physical characteristics, and physical activities associated with hamstring.^23^

**Fig 4:**
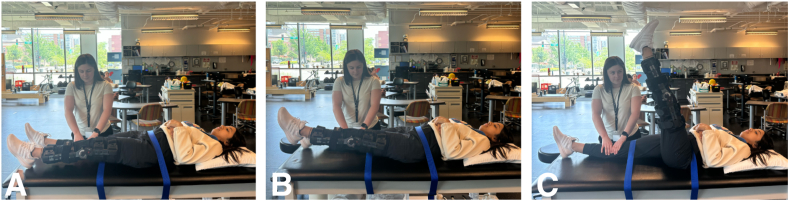
Askling H-Test. Images A through C depict the Askling H-Test. (A) The athlete starts with the hips and noninjured leg strapped to the table to isolate the injured leg’s range of motion. The knee of the injured leg is fixed in a brace to ensure extension of the leg. The physical therapist performing the test is positioned on the opposite side of the injured leg, applying downward pressure to the unaffected leg. (B) The athlete is instructed to raise the leg slightly in preparation for unilateral hip flexion. (C) The athlete is instructed to flex their injured leg explosively to 90° and quickly bring the leg back down. The athlete is asked to rate their insecurity and pain to determine apprehension.

Apprehension can be measured by the Askling H-test, which is used to evaluate an athlete’s apprehension during rapid hamstring lengthening by performing explosive unilateral hip flexion with the knee fixed in extension by a brace.^1^ An electric goniometer can also be used to quantify hip-flexion ROM during the Askling H-test, which may identify deficits that are otherwise undetected via clinical examinations of hamstring flexibility during the later stages of hamstring injury rehabilitation.^1^ Implementing the Askling H-test (Fig 4) as a final return to sport criterion is associated with a low risk of reinjury but prolonged hamstring injury rehabilitation time, and practitioners may need to consider which outcome is a higher priority for each athlete.^1^

Finally, eccentric HS strength can be objectively tested using several tools, including isokinetic dynamometry and handheld dynamometry, or during the Nordic hamstring exercise using externally fixed load cells.^1^ The evidence for eccentric hamstring strength as a risk factor for hamstring injury is conflicting, and asymmetries after return to sport were not associated with reinjury.^1^ Eccentric hamstring strength is associated with sprint acceleration mechanics, which is important for performance in running-based sports.^1^ In addition, hamstring-to-quadriceps strength ratio is another important measure of strength, where lower ratios have been associated with hamstring injury.^4^

### Return to Play and Reinjury Rates

In 4 studies implementing a combination of clinical assessments and performance tests as RTP criteria, mean RTP times and reinjury rates were 23 days and 34.8%, 26 days and 9.1%, 27 days and 63.3%, and 45 days and 34.8%.^24^ Mean RTP times and rates of reinjury in the 3 studies implementing isokinetic dynamometry as part of RTP decision-making were 12 days and 6.25%, 15 days and 13.9%, and 25 days and 9.6%.^24^ In the 2 studies implementing the Askling H-test as RTP criteria, mean time taken to RTP and rates of reinjury were 63 days and 3.6% and 36 days and 1.3%.^24^

## Surgical Technique

The common guidelines recommend that acute complete proximal hamstring avulsions/ruptures with loss of function (grade III), 2-tendon tears with retraction of 2 cm or greater, or 3-tendon tears be operated on as soon as possible.^14^^,^^19^ It has been found that in these high-grade hamstring avulsions, surgical repair leads to significantly better subjective outcomes, greater strength/endurance, and greater rate of return to preinjury sports level compared with nonsurgical management.^19^ For athletes, there are more aggressive recommendations with reattachment of isolated tendon avulsions with a structured postoperative rehabilitation program after surgery.^19^

The gold standard surgical treatment for hamstring tears uses a suture anchor system, which can be completed with an open or endoscopic approach, with the latter being more frequently employed for chronic hamstring tears.^13^ Most commonly in acute injuries, surgical technique includes a transverse incision made within the gluteal crease above the ischial tuberosity where the lower edge of the gluteus maximus muscle is exposed, retracted proximally, and the posterior cutaneous femoral nerve identified and spared.^19^ Once the retracted tendon stumps are identified, the sciatic nerve should be identified lateral to the proximal hamstring tendons and dissected from its surrounding tissue.^13^ Then, the retracted tendon stumps are captured with sutures, and the muscles are released as far distally as possible with the aim of mobilization of the stumps to their respective areas of origin on the ischium.^19^ Once the muscular origin of the ischium is prepared, grooves are cut into the ischial tuberosity at the site of detachment where up to 5 anchors/fiber wire sutures are placed.^13^^,^^19^ Lastly, the stumps are secured with locking whip stitches and ultimately tied down as a pulley to the suture anchors.^19^ Other surgical considerations are made for chronic repairs, which is beyond the scope of this article.

## Preventative Strategies

Preventative strategies to reduce the risk of a hamstring-related injury include addressing modifiable risk factors as previously discussed (Table 1). These include risk factors such as knee extension deficit, H/Q strength ratio, and eccentric hamstring strength, which can all be trained. It is recommended that team medical staff monitor players’ eccentric hamstring strength and H/Q strength ratio during the preseason and in-season and address deficits accordingly.^20^ Studies have shown that eccentric training reduces the incidence of hamstring injury between 56% and 70%, while also reducing limb strength asymmetry and imbalances in the H/Q strength ratio and athlete flexibility.^25^

In addition, preventive studies on the Nordic hamstring exercise, which focuses on eccentric training, have shown to reduce the risk of hamstring injuries.^16^ The preventive effect of the Nordic hamstring exercise may be attributed to its ability to increase muscle fascicle length because short hamstring fascicles are associated with an increased risk of a hamstring injury.^16^

## Conclusions

The incidence of hamstring injuries, particularly in professional baseball players, is increasing, likely due to a variety of factors including high-speed running involved in the sport itself as well as the transition from static standing to dynamic movements.^3^^,^^8^ While the incidence of hamstring injuries is increasing, a thorough examination, knowledge of modifiable risk factors, and a programmed rehabilitation program emphasizing eccentric strengthening can improve an athlete’s ability to return to sport and minimize missed days off due to injury. This article highlights the importance of increased attention to the evolving evidence surrounding hamstring-related injuries to address both players’ needs and wellness as well as the large competitive and financial interest for the professional baseball organizations.^7^

## Disclosures

The authors declare the following financial interests/personal relationships which may be considered as potential competing interests: S.N. has a consulting or advisory relationship with Stryker and is a board member of the American Orthopaedic Society for Sports Medicine and Arthroscopy Association of North America. All other authors (A.G., D.M., E.H., J.C.) declare that they have no known competing financial interests or personal relationships that could have appeared to influence the work reported in this paper.
